# Multiple opportunistic infections (pulmonary tuberculosis, *Mycobacterium avium* complex and parvovirus B19) in a single patient

**DOI:** 10.4102/sajhivmed.v23i1.1319

**Published:** 2022-01-25

**Authors:** Midhun T. John, Michelle Venter, Jenifer Vaughan, Marianne Black, Daniel Prince, Aishwarya M. Luke, Mithra John

**Affiliations:** 1 Department of Internal Medicine, Faculty of Health Sciences, University of the Witwatersrand, Johannesburg, South Africa; 2 Department of Infectious Diseases, Faculty of Health Sciences, University of the Witwatersrand, Johannesburg, South Africa; 3 Department of Clinical Haematology, Faculty of Health Sciences, University of the Witwatersrand, Johannesburg, South Africa; 4 Department of Microbiology, Faculty of Health Sciences, University of the Witwatersrand, Johannesburg, South Africa; 5 Department of Radiology, Faculty of Health Sciences, University of the Witwatersrand, Johannesburg, South Africa

**Keywords:** MAC, parvovirus B19, tuberculosis, HIV, multiple organisms

## Abstract

**Introduction:**

HIV infection is a common disease in the South African population. The virus can lead to the development of many opportunistic infections. This case study examines co-infection with three opportunistic infections and the need for clinical suspicion of infections in our HIV population.

**Patient presentation:**

A 36-year-old unemployed female residing in Soweto, Johannesburg, presented at Chris Hani Baragwanath Hospital (CHBAH). She was HIV positive, defaulting treatment, with no other comorbidities. She presented to CHBAH with general body weakness, diarrhoea, cough and constitutional symptoms; clinically she appeared pale and chronically ill. A differential diagnosis was made of multiple infections co-inhabiting the patient.

**Management and outcome:**

The patient had blood, sputum, radiological and invasive bone marrow aspiration, and trephine biopsies completed. The investigations revealed that she was co-infected with *Mycobacterium tuberculosis* (MTB), *Mycobacterium avium* complex (MAC) and parvovirus B19. The TB and disseminated MAC infection were managed with rifampicin, isoniazid, ethambutol, pyrazinamide and azithromycin, and reinitiation of antiretroviral (ARV) treatment was planned on further follow-up of the ARV drug resistance test. The parvovirus B19 infection was managed with immunoglobulins (Polygam) and steroids (prednisone). She was discharged successfully for further follow-up.

**Conclusion:**

A thorough history, clinical examination and subsequent targeted investigations are vital to arriving at the correct diagnosis or diagnoses. The case presented above serves to illustrate how three life-threatening opportunistic infections (OIs), all with differing treatments, may present in a single patient. Clinicians caring for immunosuppressed patients need to remain vigilant for the presence of multiple OIs occurring simultaneously.

## Introduction

Patients with a compromised immune system often present a clinical diagnostic dilemma, because they may be simultaneously infected with multiple opportunistic infections (OIs). In this case report, we present the case of a severely immunocompromised patient with advanced HIV infection, who presented with multiple simultaneous OIs.

## Case

Patient J.M. is a 36-year-old female who resides in Soweto, Johannesburg, with her family. She is unemployed and receives a government social grant.

The patient’s first presentation to Chris Hani Baragwanath Academic Hospital (CHBAH) was in November 2019. She was referred from the local clinic because of constitutional symptoms, including intermittent diarrhoea, and was reported to have anaemia.

She was diagnosed with HIV in January 2019 and defaulted on her antiretroviral treatment (ART). Her original ART regimen could not be ascertained but was restarted at a local facility a week prior to presentation. She was reinitiated onto a regimen of abacavir, lamivudine and efavirenz. Her CD4 count on first presentation was 1 cell/µL, and the HIV viral load was 29 300 copies/mL (4.47 log10 copies/mL). There were no retrievable previous trends to compare. No concomitant comorbidities were identified at the time of reinitiation.

Clinically, she was generally wasted and anaemic. She had unilateral crackles in the right middle lobe of the lung, with no respiratory distress and normal oxygen saturation. The rest of the clinical exam was unremarkable.

She was admitted from the Medical Outpatients’ Department (MOPD) to the medical wards for further investigation and management.

The patient was found to have severe normochromic anaemia with a haemoglobin level of 3.0 g/dL. The differential count revealed that she had severe aplastic pancytopenia, as evidenced by a reticulocyte production index (RPI) of 0.0%. The remaining urea, electrolytes, calcium, magnesium and phosphate were normal (see Table 1).

**TABLE 1:** Initial blood results.

Variable	Initial blood results
**Full blood count**	
White cell count	2.01 × 109/L (3.9–12.60)
Red cell count	1.21 × 1012/L (3.93–5.40)
Haemoglobin	3.0 g/dL (11.6–16.4)
Haematocrit	0.111 L/L (0.340–0.480)
Mean cell volume	91.7 fL (78.9–98.5)
Platelet count	163 × 109/L (150–300)
**Differential count**	
Neutrophils	0.73 × 109/L (1.6–8.3)
Lymphocytes	0.82 × 109/L (1.4–4.5)
**Anaemia studies**	
Iron	8.3 µmol/L (9–30.4)
Transferrin	1.31 g/L (2.5–3.8)
% saturation	25% (15–50)
Ferritin	2507 µg/L (15–150)
Vitamin B12	1476 pmol/L (141–489)
Serum folate	24.1 nmol/L (8.8–60.8)
Coomb’s test	Negative
Reticulocyte production index	0%
**Liver function tests**	
Total protein	77 g/L (60–78)
Albumin	20 g/L (35–52)
Total bilirubin	5 µmol/L (5–21)
Conjugated bilirubin	3 µmol/L (0–3)
Alanine transaminase	5 U/L (7–35)
Aspartate transaminase	21 U/L (13–35)
Alkaline phosphatase	188 U/L (42–98)
Gamma-glutamyl transferase	116 U/L (< 40)
Lactate dehydrogenase	284 U/L (100–190)

During this admission, her sputum was submitted for GeneXpert (GXP) mycobacterium/rifampicin (MTB/RIF) Ultra testing (Cepheid) and was positive for *Mycobacterium tuberculosis* complex (rifampicin susceptible). She was initiated onto antituberculosis treatment and infused blood products and was discharged for outpatient follow-up for review of all her investigations, including a bone marrow aspirate and trephine (BMAT).

The patient presented to the MOPD a month later (January 2020), complaining of abdominal pain, diarrhoea and dizziness for two weeks. She was readmitted for investigation and management. Again, pancytopenia was noted (Table 2).

**TABLE 2:** Follow-up blood results.

Variable	Follow-up blood results
**Full blood count**	
White cell count	2.06 × 10^9^/L (3.9–12.60)
Red cell count	1.01 × 10^12^/L (3.93–5.40)
Haemoglobin	2.7 g/dL (11.6–16.4)
Haematocrit	0.087 L/L (0.340–0.480)
Mean cell volume	86.1 fL (78.9–98.5)
Mean corpuscle haemoglobin concentration	31.0 pg (26.1–33.5)
Red cell distribution width	16.4% (12.4–17.3)
Platelet count	113 × 10^9^/L (7.3–11.3)
**Differential count**	
Neutrophils	0.73 × 109/L (1.6–8.3)
Lymphocytes	0.82 × 109/L (1.4–4.5)
**Liver function tests**	
Alkaline phosphatase	188 U/L (42–98)
Gamma-glutamyl transferase	116 U/L (< 40)
Lactate dehydrogenase	284 U/L (100–190)

Because of her clinical deterioration, despite treatment for microbiologically confirmed tuberculosis (TB), other OIs and the possibility of drug-resistant TB were suspected, and a further workup was initiated.

**FIGURE 1:**
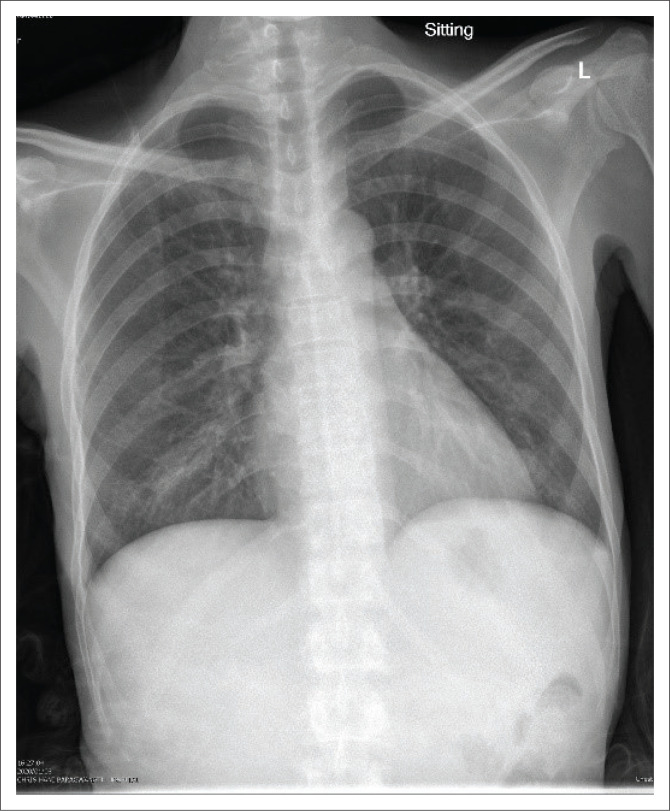
Chest radiograph on patient on readmission (1-month post-antituberculosis treatment). Patchy consolidation of the right upper lobe, as well as the right and left lower lobes. Silhouetting of the right heart border. These are features in keeping with bronchopneumonia with right middle lobe consolidation and/or atelectasis.

The initial chest radiograph could not be retrieved, but a chest radiograph taken after one month of antituberculosis treatment showed features in keeping with bronchopneumonia, with right middle lobe consolidation and/or atelectasis (see Figure 1).

**FIGURE 2:**
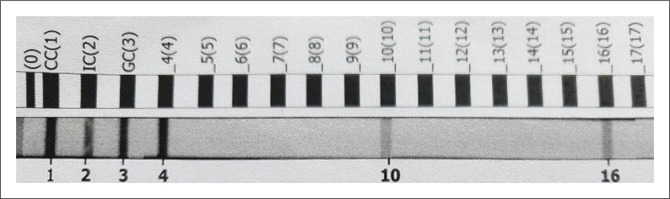
Line probe assay (GenoType *Mycobacterium* CM version 2.0) for patient J.M., from sputum culture, collected 18/11/2019. The conjugate control (CC), internal control (IC) and genus control for mycobacteria (GC) is positive (lines numbered 1, 2, 3). *Mycobacterium avium* is identified (line 4), as well as *Mycobacterium tuberculosis* complex (lines 10 and 16).

The mycobacterial culture from the sputum specimen collected on the first admission flagged positive in 11 days. *Mycobacterium avium*, as well as *M. tuberculosis* complex, was identified using the GenoType *Mycobacterium* CM version 2.0 (Hain Lifescience, Centurion, South Africa) line probe assay (see Figure 2). The line probe assay for susceptibility testing for *M. tuberculosis* complex, MTBDRplus version 2.0 (Hain Lifescience) was unsuccessful, because there were mixed mycobacterial species present.

In addition, *M. avium* complex (MAC) was identified on a mycobacterial blood culture (BACTEC Myco/F Lytic bottle; Becton Dickinson), collected on the first admission, confirming the diagnosis of disseminated MAC infection (see Table 3).

**TABLE 3:** Microbiological investigations for mycobacterial infection.

Specimen type	Date of specimen collection	Molecular assay for mycobacterial identification	Species identified
Blood culture	11/11/2019	GenoType *Mycobacterium* CM version 2.0	*M. avium*
Sputum	18/11/2019	GenoType *Mycobacterium* CM version 2.0	*M. tuberculosis* complex and *M. avium*
Sputum	18/11/2019	Xpert MTB/RIF Ultra	*M. tuberculosis* complex, rifampicin susceptible

**FIGURE 3:**
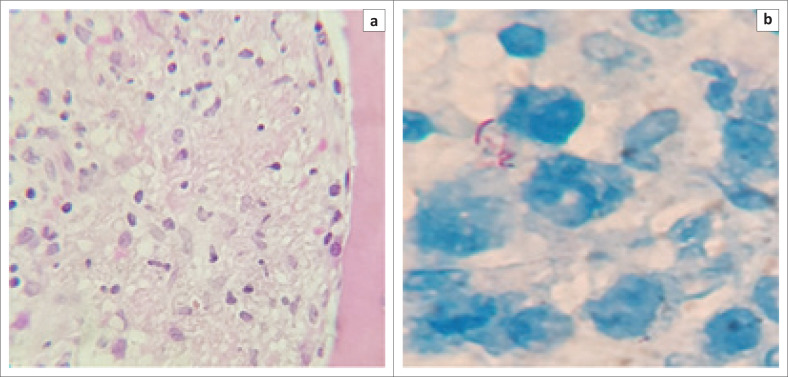
Bone marrow trephine biopsy showing acid-fast bacilli. (a) An area of granulomatous inflammation (haematoxylin and eosin, 200× magnification). (b) A Ziehl-Neelson stain was positive for scanty acid-fast bacilli (black arrow, 1000× magnification).

The BMAT performed during her first admission showed granulomatous inflammation with a Ziehl-Neelsen stain positive for acid-fast bacilli (AFB) (see Figure 3).

**FIGURE 4:**
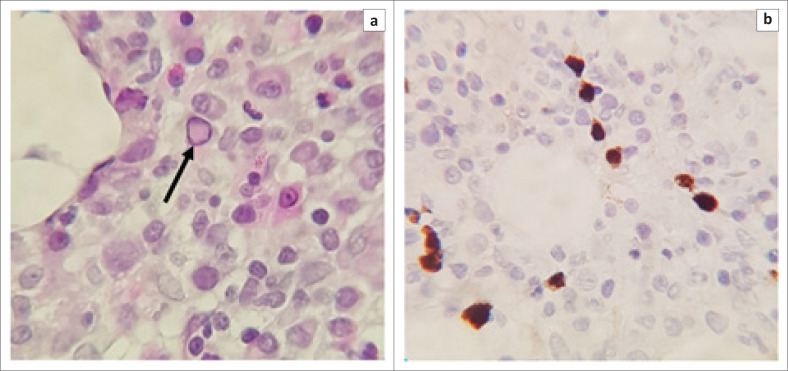
Bone marrow trephine biopsy showing parvovirus B-19 Inclusions. (a) Occasional parvovirus inclusions were seen (black arrow, haematoxylin and eosin, 500× magnification). (b) Parvovirus immunohistochemical stain positive (brown intranuclear staining, 400× magnification).

On the BMAT, a pure red cell aplasia (PRCA) was noted, attributable to parvovirus B19 infection. On peripheral blood, the patient still had an RPI of 0.0% and antibodies for parvovirus flagged for current infection, supporting the diagnosis of a concomitant PRCA, most likely due to parvovirus B19 co-infection (see Figure 4).

The patient presented with three microbiologically and pathologically confirmed OIs: pulmonary MTB, disseminated MAC infection and parvovirus B19 infection. The parvovirus B19 infection was managed with intravenous immunoglobulin for three days, blood products and oral steroids. The patient’s blood count improved.

The TB and disseminated MAC infection were managed with rifampicin, isoniazid, ethambutol, pyrazinamide and azithromycin, and ART was placed on hold pending the results of the antiretroviral (ARV) drug resistance test. The plan was to reinitiate ART after four weeks.

The patient was subsequently discharged after three weeks of admission and was clinically stable. She was planned for follow-up at both the Haematology and Infectious Diseases clinics.

Unfortunately, the patient did not return for her follow-up visits; she relocated to another province and was lost to follow-up.

## Discussion

Careful history taking, examination and the appropriate use of investigations are crucial in identifying concomitant OIs in immunosuppressed patients.

According to the World Health Organization, more than 10 million people were infected with TB in 2018. Of those patients infected with TB, 1.5 million people have died.^1^ The risk of acquiring TB in the setting of HIV is 9–16 times that of an HIV-uninfected individual.^2^ In the above case, the patient’s constitutional symptoms and the investigation of her sputum and blood cultures confirmed mycobacterial infection, MAC infection as well as parvovirus B19.

The overall prevalence of parvovirus B19 is likely to be highly underestimated, as it may only become clinically apparent during an episode of reactivation.^3^ In our setting of HIV, bone marrow aspirate is the method of choice to diagnose co-infection.^4^

Non-tuberculous mycobacteria (NTM) species, such as MAC, are seen more commonly in patients with CD4 counts of < 50 cells/mL.^5^ The risk of developing MAC infection is increased with other concurrent infections such as TB.^6^

Along with these clinical features and a positive mycobacterial blood culture, laboratory features supporting a diagnosis of disseminated MAC infection include a raised alkaline phosphatase and gamma-glutamyl transferase.^7^ A diagnosis of MAC infection will be missed if only the GXP assay is requested, because this assay detects the *M. tuberculosis* complex only.

Blood culture is the preferred initial test; however, there are a few limitations that need to be noted.^8^ In local MAC infection, blood cultures are negative.^9^ Although MAC is classified as a fast-growing NTM, the culture may only become positive after 1–2 weeks.^9^ Hussong et al.^9^ reported that blood cultures, bone marrow aspirate cultures, AFB stains and granuloma detection complement the investigations well. Acid-fast bacillus staining often has the fastest detection rate of disseminated MAC infection, and if positive, it triggers prompt anti-MAC treatment.^9^

## Management dilemma

The management of MAC consists of a multidrug regimen of different antimicrobials.^10^ Macrolides (azithromycin and clarithromycin) are the mainstay treatment for MAC and need to be used with one or more agents to reduce the potential of drug-resistant MAC infections.^10^

The patient in our study would have been placed on an oral regimen of azithromycin (500 mg tablet daily), ethambutol (15 mg/kg daily) and rifabutin (300 mg daily).^10^ However, this was not the case because of her co-infection with pulmonary tuberculosis and drug-resistant HIV.

The treatment duration for MAC infection is 12 months after the first negative sputum culture.

## Conclusion

Treating the immunocompromised patient is not an easy task. The case presented above serves to illustrate how three life-threatening OIs, all with differing treatments, may present in a single patient. Clinicians caring for patients with suppressed immune systems are urged to remain vigilant for the presence of multiple OIs occurring simultaneously.
